# Complete Genome Sequence of *Methanothermobacter* sp. Strain THM-1, a Thermophilic and Hydrogenotrophic Methanogen Isolated from an Anaerobic Reactor in South Korea

**DOI:** 10.1128/MRA.00587-21

**Published:** 2021-09-23

**Authors:** Byoung-Seung Jeon, Hyunjin Kim, Young Wook Go, Hyunook Kim, Okkyoung Choi, Byoung-In Sang

**Affiliations:** a Convergence Bioceramic Materials Center, Korea Institute of Ceramic Engineering and Technology, Cheongju, Republic of Korea; b Department of Chemical Engineering, Hanyang University, Seoul, Republic of Korea; c Department of Environmental Engineering, University of Seoul, Seoul, Republic of Korea; University of Southern California

## Abstract

*Methanothermobacter* sp. strain THM-1, a thermophilic and hydrogenotrophic methanogen, was isolated from an anaerobic reactor enriched with thermophilic methanogens. The genome of THM-1 shares 98.81% of its sequence with Methanothermobacter wolfeii isolate SIV6 and consists of 1,724,502 bp with 1,665 protein-coding genes, 50 noncoding RNAs, and a GC content of 48.6%.

## ANNOUNCEMENT

Thermophilic and hydrogenotrophic methanogens (THMs) have been suggested to produce methane from carbon dioxide and hydrogen in power-to-gas and biogas upgrading processes (1).

Here, we report the complete genome sequence of the newly isolated methanogen *Methanothermobacter* sp. strain THM-1 (hereinafter THM-1). THM-1 was isolated from an anaerobic reactor integrated with a bioelectrochemical system, which produced methane and acetate from carbon dioxide using electricity (2). Less than 10 μl of the reactor sample was injected into 50 ml of basic anaerobic agar (1.5% [wt/vol] agar added to basic anaerobic medium [2]) reinforced with sodium bicarbonate. The agar solidified after homogenization at approximately 60°C (3, 4). The anaerobic agar medium containing the bacteria was incubated at 60°C under atmospheric conditions that included CO_2_ and H_2_ at a ratio of 1:4. The samples were cultivated until 1- to 2-mm colonies were visible inside the agar medium. The agar, including a single colony, was then diced into 0.125-cm^3^ pieces and sequentially subcultured. Identification by amplification and sequencing of the 16S rRNA gene was conducted using the primers from a previous study (5). THM-1 was classified as a member of the genus Methanothermobacter by phylogenetic comparison of the 16S rRNA genes using MEGA7 (6, 7).

The DNA of THM-1 was taken from cells purely cultivated, which were enriched in basic anaerobic medium at 60°C under a pressurization system of 2 bars. In this system, CO_2_ and H_2_ were used as the sole carbon and energy sources, respectively. Genomic DNA was extracted using the DNeasy UltraClean microbial kit (Qiagen Korea Ltd., South Korea) (8) and sequenced using the PacBio RS II platform (PacBio single-molecule real-time sequencing) at Macrogen Co. Ltd. (Seoul, South Korea). The library was prepared using the SMRTbell template prep kit (9). The preassembly read quality check (QC) was performed using a BLAST v2.7.1 search of the subreads with the NCBI nucleotide database, excluding the phage sequences (10). *De novo* assembly was performed using Falcon-integrate v2.1.4 (11). Default parameters were used for all software unless otherwise specified. After the whole genome was assembled, the gene functions were annotated using the NCBI Prokaryotic Genome Annotation Pipeline (12).

The filtered data included a total of 1,283,479,478 bases, and the number of reads was 139,486. The mean subread and *N*_50_ read lengths were 9,201 bases and 14,093 bases, respectively. The genome comprised a single chromosome (1,724,502 bp) with an average reference coverage of 533×. One contig was generated, and both ends of the contig overlapped, indicating that the genome is circular. The genes from the assembled genome were annotated, and a total of 1,808 genes were identified, of which 1,665 were predicted to contain coding sequences (CDSs); 39 were tRNAs, and 9 were rRNAs. The GC content was 48.59%. The orthologous average nucleotide index (OrthoANI) of THM-1 was 98.81% of the nucleotide sequence of Methanothermobacter wolfeii isolate SIV6 (13, 14). The OrthoANI was measured using OAT v0.93 (15). The shuttle-vector system for Methanothermobacter species was recently developed (16). The genetic resources for THMs obtained via genome sequencing could be applied to develop highly effective methanogenic archaea to utilize CO_2_ and convert it into energy. This resource is expected to accelerate global efforts to reduce carbon dioxide.

### Data availability.

This whole-genome project (in GenBank format) and the corresponding raw data files (fastq format) have been deposited under the GenBank accession number CP044013 and the SRA accession number SRR10567007, respectively. The 16S rRNA gene was deposited under the GenBank accession number MH605439.

## References

[1] Vo TTQ, Xia A, Wall DM, Murphy JD. 2017. Use of surplus wind electricity in Ireland to produce compressed renewable gaseous transport fuel through biological power to gas systems. Renew Energy 105:495–504. doi:10.1016/j.renene.2016.12.084.

[2] Song H, Choi O, Pandey A, Kim YG, Joo JS, Sang B-I. 2019. Simultaneous production of methane and acetate by thermophilic mixed culture from carbon dioxide in bioelectrochemical system. Bioresour Technol 281:474–479. doi:10.1016/j.biortech.2019.02.115. 30853369

[3] Angelidaki I, Sanders W. 2004. Assessment of the anaerobic biodegradability of macropollutants. Rev Environ Sci Biotechnol 3:117–129. doi:10.1007/s11157-004-2502-3.

[4] Choi O, Kim M, Go Y, Hong M-G, Kim B, Shin Y, Lee S, Kim YG, Joo JS, Jeon BS, Sang B-I. 2019. Selective removal of water generated during hydrogenotrophic methanation from culture medium using membrane distillation. Energies 12:4130. doi:10.3390/en12214130.

[5] Jeon BS, Kim H, Go YW, Kim YG, Joo JS, Choi O, Sang B-I. 2020. Complete genomic sequence of the thermophilic and hydrogenotrophic methanogen *Methanothermobacter* sp. strain KEPCO-1. Microbiol Resour Announc 9:e01034-19. doi:10.1128/MRA.01034-19. 31896626 PMC6940278

[6] Stackebrandt E, Goebel BM. 1994. Taxonomic note: a place for DNA-DNA reassociation and 16S rRNA sequence analysis in the present species definition in bacteriology. Int J Syst Evol Microbiol 44:846–849. doi:10.1099/00207713-44-4-846.

[7] Kumar S, Stecher G, Tamura K. 2016. MEGA7: Molecular Evolutionary Genetics Analysis version 7.0 for bigger datasets. Mol Biol Evol 33:1870–1874. doi:10.1093/molbev/msw054. 27004904 PMC8210823

[8] Göttig S, Frank D, Mungo E, Nolte A, Hogardt M, Besier S, Wichelhaus TA. 2019. Emergence of ceftazidime/avibactam resistance in KPC-3-producing *Klebsiella pneumoniae in vivo*. J Antimicrob Chemother 74:3211–3216. doi:10.1093/jac/dkz330. 31365094

[9] Rhoads A, Au KF. 2015. PacBio sequencing and its applications. Genomics Proteomics Bioinformatics 13:278–289. doi:10.1016/j.gpb.2015.08.002. 26542840 PMC4678779

[10] Camacho C, Coulouris G, Avagyan V, Ma N, Papadopoulos J, Bealer K, Madden TL. 2009. BLAST+: architecture and applications. BMC Bioinformatics 10:421. doi:10.1186/1471-2105-10-421. 20003500 PMC2803857

[11] Chin C-S, Peluso P, Sedlazeck FJ, Nattestad M, Concepcion GT, Clum A, Dunn C, O’Malley R, Figueroa-Balderas R, Morales-Cruz A, Cramer GR, Delledonne M, Luo C, Ecker JR, Cantu D, Rank DR, Schatz MC. 2016. Phased diploid genome assembly with single-molecule real-time sequencing. Nat Methods 13:1050–1054. doi:10.1038/nmeth.4035. 27749838 PMC5503144

[12] Li W, O’Neill KR, Haft DH, DiCuccio M, Chetvernin V, Badretdin A, Coulouris G, Chitsaz F, Derbyshire MK, Durkin AS, Gonzales NR, Gwadz M, Lanczycki CJ, Song JS, Thanki N, Wang J, Yamashita RA, Yang M, Zheng C, Marchler-Bauer A, Thibaud-Nissen F. 2021. RefSeq: expanding the Prokaryotic Genome Annotation Pipeline reach with protein family model curation. Nucleic Acids Res 49:D1020–D1028. doi:10.1093/nar/gkaa1105. 33270901 PMC7779008

[13] Kim M, Oh H-S, Park S-C, Chun J. 2014. Towards a taxonomic coherence between average nucleotide identity and 16S rRNA gene sequence similarity for species demarcation of prokaryotes. Int J Syst Evol Microbiol 64:346–351. doi:10.1099/ijs.0.059774-0. 24505072

[14] Hassa J, Wibberg D, Maus I, Pühler A, Schlüter A. 2020. Genome analyses and genome-centered metatranscriptomics of Methanothermobacter wolfeii strain SIV6, isolated from a thermophilic production-scale biogas fermenter. Microorganisms 8:13. doi:10.3390/microorganisms8010013. PMC7022856 31861790

[15] Lee I, Kim YO, Park S-C, Chun J. 2016. OrthoANI: an improved algorithm and software for calculating average nucleotide identity. Int J Syst Evol Microbiol 66:1100–1103. doi:10.1099/ijsem.0.000760. 26585518

[16] Fink C, Beblawy S, Enkerlin AM, Mühling L, Angenent LT, Molitor B. 2021. A shuttle-vector system allows heterologous gene expression in the thermophilic methanogen *Methanothermobacter thermautotrophicus* ΔH. bioRxiv doi:10.1101/2021.04.20.440605. PMC8609365 34809461

